# Supplementary material for: Complexome profiling of the Chlamydomonas psb28 mutant reveals TEF5 as an early PSII assembly factor
Source: Plant Cell. 2025 Mar 17;37(6):koaf055. doi: 10.1093/plcell/koaf055 (PMC12164590; doi:10.1093/plcell/koaf055)
Supplement: koaf055_Supplementary_Data [file koaf055_supplementary_data.zip › psb28_interactive.zip!/plots/intensity/qualityPlots/peptides/NCR2_Cre01.g039350.t1.2.html]

### Cre01.g039350.t1.2

Trivial name: NCR2  
SeqCoverage [median]: 4.90  
Mass: 70.40 kDa  
  
 

| Sequence | MissedCleavage | Mass [Da] | wtIntensity | muIntensity | Score |
| --- | --- | --- | --- | --- | --- |
| ILYGTQTGTAER | 0 | 1308.67 | 0.00 | 460.27 | 64.73 |
| LALLGSPAGK | 0 | 925.56 | 0.00 | 889.04 | 50.04 |
| VAAYEVEVLR | 0 | 1147.62 | 0.00 | 1523.84 | 74.46 |
|  |  |  | 0.0 | 2873.1 |  |
